# Diversity and distribution of parasitic angiosperms in China

**DOI:** 10.1002/ece3.3992

**Published:** 2018-04-02

**Authors:** Guangfu Zhang, Qian Li, Shucun Sun

**Affiliations:** ^1^ Jiangsu Key Laboratory of Biodiversity and Biotechnology School of Life Sciences Nanjing Normal University Nanjing China; ^2^ College of Life Sciences Nanjing University Nanjing China

**Keywords:** China, hemiparasites, holoparasites, life forms, parasitic angiosperm, species richness

## Abstract

Parasitic plants are an important component of vegetation worldwide, but their diversity and distribution in China have not been systematically reported. This study aimed to (1) explore floral characteristics of China's parasitic plants, (2) map spatial distribution of diversity of these species, and (3) explore factors influencing the distribution pattern. We compiled a nationwide species list of parasitic plants in China, and for each species, we recorded its phylogeny, endemism, and life form (e.g., herb vs. shrub; hemiparasite vs. holoparasite). Species richness and area‐corrected species richness were calculated for 28 provinces, covering 98.89% of China's terrestrial area. Regression analyses were performed to determine relationships between provincial area‐corrected species richness of parasitic plants and provincial total species richness (including nonparasitic plants) and physical settings (altitude, midlongitude, and midlatitude). A total of 678 species of parasitic angiosperms are recorded in China, 63.13% of which are endemic. Of the total, 59.73% (405 species) are perennials, followed by shrubs/subshrubs (14.75%) and vines (1.47%). About 76.11% (516 species) are of root hemiparasites, higher than that of stem parasites (100, 14.75%), root holoparasites (9.00%), and endophytic parasites (0.15%). A significant positive relationship is found between the area‐corrected species richness and the total species richness, which has been previously demonstrated to increase with decreasing longitude and latitude. Moreover, more parasitic species are found in the southwest high‐altitude areas than low areas. Consistently, the area‐corrected species richness increases with increasing altitude, decreasing latitude, and decreasing longitude, as indicated by regression analyses. China is rich in parasitic flora with a high proportion of endemic species. Perennials and root hemiparasites are the dominant types. The spatial distribution of parasitic plants is largely heterogeneous, with more species living in southwest China, similar to the distribution pattern of Chinese angiosperms. The positive relationship between parasitic and nonparasitic plant species richness should be addressed in the future.

## 1 INTRODUCTION

Parasitic plants are a particular functional species group, which form haustoria obtaining water and nutrients, as well as carbohydrate, partly or wholly from their hosts (Poulin, 2011). They are an important component of vegetation worldwide, influencing ecosystem structure and function (Bardgett et al., 2006; Li & Dong, 2011; Těšitel et al., 2017). For example, parasitic plants may confer profound effects on population dynamics across different trophic levels because of the differences in plant traits (e.g., leaf, flower, and fruit phenology and property) with their hosts (Hartley et al., 2015). As such, they are sometimes considered as community key stone species and ecosystem engineers (Press & Phoenix, 2005). Studies have extensively investigated phylogenetic evolution of parasitic plants (Bromham, Cowman, & Lanfear, 2013; Calatayud et al., 2016), host–parasite interactions (Brown & Tellier, 2011; Calatayud et al., 2016; Dueholm et al., 2017; Grewell, 2008), and possible harmful effects on crop production (Fernández‐Aparicio, Reboud, & Gibot‐Leclerc, 2016; Sauerborn, Mullerstover, & Hershenhorn, 2007; Zwanenburg, Mwakaboko, & Kannan, 2016). However, the diversity and distribution of parasitic plants have received much less attention relative to those of particular plant taxa (e.g., parasitic angiosperms in a certain region; Joel, Gressel, & Musselman, 2013; Kavanagh & Burns, 2012; Santos, Nascimento, Marzinek, & Leiner, 2017).

There is a high species diversity of parasitic plants partly because of a higher mutation rate compared to their nonparasitic relatives (Bromham et al., 2013). It has been estimated that there are ~4,500 parasitic plant species, accounting for about 1% of the whole angiosperms in the world (Heide‐Jørgensen, 2008). Moreover, parasitic species are not derived from a monophyletic group, and they have independently evolved at least 12 times (Bellot & Renner, 2013; Naumann et al., 2013; Westwood, Yoder, Timko, & Depamphilis, 2010), which indicate that parasitic plants could be diverse both evolutionally and ecologically. Consistently, parasitic plants are widely distributed in various natural and seminatural ecosystems ranging from tropical rain forest to Arctic tundra, and moreover, they differ in life forms, for example stem vs. leaf parasite and hemiparasite vs. holoparasite (Poulin, 2011; Stewart & Press, 1990). Several reports have recorded the species richness at the national level (e.g., 151 parasitic angiosperms in Nepal, O'Neill & Rana, 2016; 146 in Turkey, Sürmen, Kutbay, & Yilmaz, 2015). Nevertheless, the diversity of parasitic angiosperms, as well as the factors influencing the diversity, has seldom been well recorded for countries with large terrestrial area and high species richness.

Besides, there is little research which deals with the factors contributing to geographical pattern of parasitic angiosperms across different climates. Watson (2009) proposed “the host‐quality hypothesis” to account for nonrandom distribution pattern of parasitic plants. Joel et al. (2013) pointed out that the majority of parasites had a wide host range, which was mainly influenced by host geographical distribution and ecological relationships. Luo, Sui, Gan, and Zhang (2015) contended that host compatibility interacting with seed dispersal determined small‐scale distribution of the mistletoe *Dendrophthoe pentandra* (Loranthaceae) in Xishuangbanna, southwest China. In fact, the distribution of a parasitic plant flora in a certain area generally results from biological (i.e., dispersal vector and host availability) and environmental factors (i.e., altitude, area, longitude, and latitude).

China has a large terrestrial area of 9.60 million km^2^ and is the third largest country in the world. China's territory stretches 5,200 km from east to west and 5,500 km from south to north (ECCPG, 1985a), ranging between tropical, subtropical, warm‐temperate, temperate, and cold‐temperate biome. Because of a wide range of climate, combined with highly complex topography and wide range of habitats, China has a tremendous diversity of plant and animal species (Wu, 1980; Zhang, 2007a,b), with a recent record of ~34,450 indigenous higher plant species (Zhao, Li, Liu, & Qin, 2016). Assuming that parasite species richness is proportional to host species diversity, we may speculate that the spatial pattern of parasite species richness is similar to the general pattern of Chinese plant diversity. Additionally, the distribution of local species richness of Chinese plants is well known to be affected by climatic conditions including annual mean temperature and precipitation. Specifically, plant species diversity generally increases with increasing mean annual temperature (MAT) and mean annual precipitation (MAP) within China. Thus, we may further speculate that the species richness distribution of parasitic plants should present a similar relationship with climatic conditions.

In this study, we compiled the most comprehensive checklist of parasitic angiosperms throughout China, recording taxonomic status, endemism, life form, and geographical distribution for each parasitic plant species. The primary objective of this study is to characterize the floral characteristics and spatial distribution pattern of parasitic plant species richness in China. Specifically, we address the following questions: (1) How many parasitic angiosperms occur in China? And how many of them are endemic to China? (2) What are the characteristics of these plants in terms of life forms? (3) How the parasitic species are distributed in China and what contributed to distribution pattern? To the best of our knowledge, this is the first report about the diversity and distribution of parasitic angiosperms in China.

## 2 METHODS

### 2.1 Data sources

Only the species that obtain nutrients from host plants by haustorium were included in this study, according to the definition of parasitic plants by Heide‐Jørgensen (2008). We did not include epiphytes, stranglers, and mycoheterotrophic plant species (or saprophytes) because epiphytes and stranglers do not uptake water and nutrients from their hosts and mycoheterotrophic plants obtain nutrients by means of hypha rather than haustorium. We also excluded alien, cultivated, or naturalized plants. A typical example is *Santalum album*, distributed in Pacific islands, which has been widely cultivated in Guangdong and Taiwan of China for more than one thousand years (ECFRPS, 1959–2004).

Data on parasitic species were mainly collected from published literatures and floras. First, a database was initially created from two books, *Flora Reipublicae Popularis Sinicae* (ECFRPS, 1959–2004) and *Flora of China* (Wu, Raven, & Hong, 1994–2012). The former, consisting of 80 volumes, contains a comprehensive list of Chinese vascular plants. The latter, consisting of 25 volumes, is the English revision of the former. We searched for such words as “parasit*,” “hemiparasit*,” and “holoparasit*” in English or in Chinese from the books. If a species’ description contains one of these words, the species was considered as parasitic. Then, its taxonomic status (family and genus, species, subspecies, varieties, or forms), functional group type (root hemiparasites, root holoparasites, stem parasites, and endophytic parasites; Těšitel, 2016), life form (herb/shrub), endemism (native/alien), and distribution location (Provinces within China) were recorded. In particular, we assigned *Cuscuta* species as holoparasites, because they have no roots and their leaves are too small to contribute significant photosynthetic carbohydrate to plants (McNeal, Arumugunathan, Kuehl, Boore, & dePamphilis, 2007; Těšitel, 2016). We also referred to published literature (e.g., research articles, local floras, monographs, collections, and reports; Ding, Li, Fu, & Yang, 2010; Joel et al., 2013; Liu, 2013–2016; Li & Ding, 2005; Zhang, 2007a,b), as well as websites (http://www.cvh.ac.cn; http://foc.eflora.cn/) to update the checklist. For example, *Monochasma savatieri*, a root hemiparasite indeed, was recorded by Zhang et al. (2015) and hence was amended to the checklist, although it was not mentioned elsewhere. After sorting out the checklist of parasitic angiosperms, we arranged all families and genera according to APG IV (2016). The final version of the checklist was shown in Table S1.

China is officially consisted of 32 provinces/autonomous regions (minority‐dominated regions)/municipalities. According to the studies addressing biological diversity (Huang, Chen, Ying, & Ma, 2011; Weber, Sun, & Li, 2008), the municipalities Beijing and Tianjin were emerged into Hebei Province, and the municipalities Shanghai and Chongqing were merged into Jiangsu Province and Sichuan Province, respectively. Therefore, we created 28 units (provinces/autonomous regions/municipalities) in this study, covering 98.89% terrestrial area of China. For each unit, the number of native angiosperms, area, altitude, midlongitude, and midlatitude were derived from geographical data based on *Diversity and geographic distribution of endemic species of seed plants in China* (Huang, Ma, & Chen, 2014).

### 2.2 Data analyses

We first calculated area‐corrected species richness of parasitic plants from the raw species number (species richness) for each provincial unit as *D *= *N*/log (*A*), where *N* is the number of parasitic species and *A* is the unit area (Rejmánek & Randall, 1994; Xing, Zhang, Fan, & Zhao, 2016). Then, we conducted linear regression analyses to determine the relationships between log‐scale parasitic area‐corrected species richness and physical setting (altitude, midlongitude, and midlatitude) and also log‐scale total species number within the unit (total species richness). Moreover, multiple regression analyses were carried out to determine the primary factors (among altitude, midlongitude, and midlatitude) on the area‐corrected species richness across China (Xue, 2011). All statistical analyses were performed using SPSS 22.0 (SPSS Inc., Chicago, IL, USA) and ORIGIN 8.6 (Origin Laboratory Corporation, Northampton, MA, USA).

## 3 RESULTS

### 3.1 Floristic composition

A total of 678 eudicotyledonous parasitic angiosperms belonging to 12 families and 50 genera were recorded in China, accounted for 2.28% of angiosperm species nationwide (29,716 species, Wang, Jia, Zhang, & Qin, 2015) and 15.07% of parasitic species worldwide (Table 1). No fern or gymnosperm parasite was found in China. Three families, Orobanchaceae (*n* = 530), Loranthaceae (*n* = 61), and Santalaceae (*n* = 52), occupied ca. 94.84% of the total number of parasitic plant species in China (78.17%, 9.00%, and 7.67%, respectively), while each of the rest families accounted for <2.00%.

**Table 1:** Species richness and endemism of China's parasitic plants

Family	Genus	Species	Total species (%)	Species endemic to China	Endemic species per family (%)
Orobanchaceae	22	530	78.17	379	71.51
Loranthaceae	8	61	9.00	23	37.70
Santalaceae	9	52	7.67	21	40.38
Balanophoraceae	2	13	1.92	1	7.69
Convolvulaceae	1	13	1.92	3	23.08
Mitrastemonaceae	1	2	0.29	1	50.00
Opiliaceae	2	2	0.29	–	–
Cynomoriaceae	1	1	0.15	–	–
Lauraceae	1	1	0.15	–	–
Paulowniaceae	1	1	0.15	–	–
Rafflesiaceae	1	1	0.15	–	–
Schoepfiaceae	1	1	0.15	–	–
Total	50	678	100.00	428	–

Among the parasitic plant species, 428 species belonging to 22 genera of six families were endemic, accounting for 63.13% of the total number of parasitic plant species (Table 1). Orobanchaceae included the largest number of parasitic species (*n* = 379), followed by Loranthaceae (*n* = 23) and Santalaceae (*n* = 21). With the exception of Balanophoraceae (7.69%), each of these families contained more than 20.00% of endemic species.

### 3.2 Life forms

Most of the parasitic species were herbs (*n* = 568, 83.78%), followed by shrubs (*n* = 89, 13.13%), subshrubs (*n* = 11, 1.62%), and lianas (*n* = 10, 1.47%; Table 2). Of the herbaceous parasites, 405 species were perennials and 142 species were annuals, whereas only 21 species were biennials.

**Table 2:** Life form of parasitic plants in China

Life form	Herb				Shrub	Subshrub	Vine/Liana	Total
	Annual	Biennial	Perennial	Subtotal				
Number of species	142	21	405	568	89	11	10	678
Percentage of total species (%)	20.94	3.10	59.73	83.78	13.13	1.62	1.47	100.00

The ecotypes of the parasites could be divided into four categories: root hemiparasites (e.g., *Pedicularis* spp.), root holoparasites (e.g., *Balanophora* spp.), stem parasites (e.g., *Taxillus* spp.), and endophytic parasites (e.g., *Sapria himalayana*; Table 3). Most of the parasitic species belonged to root hemiparasites (*n* = 516, 76.11% of the total), followed by stem parasites (*n* = 100, 14.75%), root holoparasites (*n* = 61, 9.00%), and endophytic parasites (*n* = 1, 0.15%).

**Table 3:** Type of parasitic plants in China

Type of parasites	Root hemiparasites	Root holoparasites	Stem parasites	Endophytic parasites	Total
No. of species	516	61	100	1	678
Total species (%)	76.11	9.00	14.75	0.15	100.00

### 3.3 Geographical distributions

**Figure 1:**
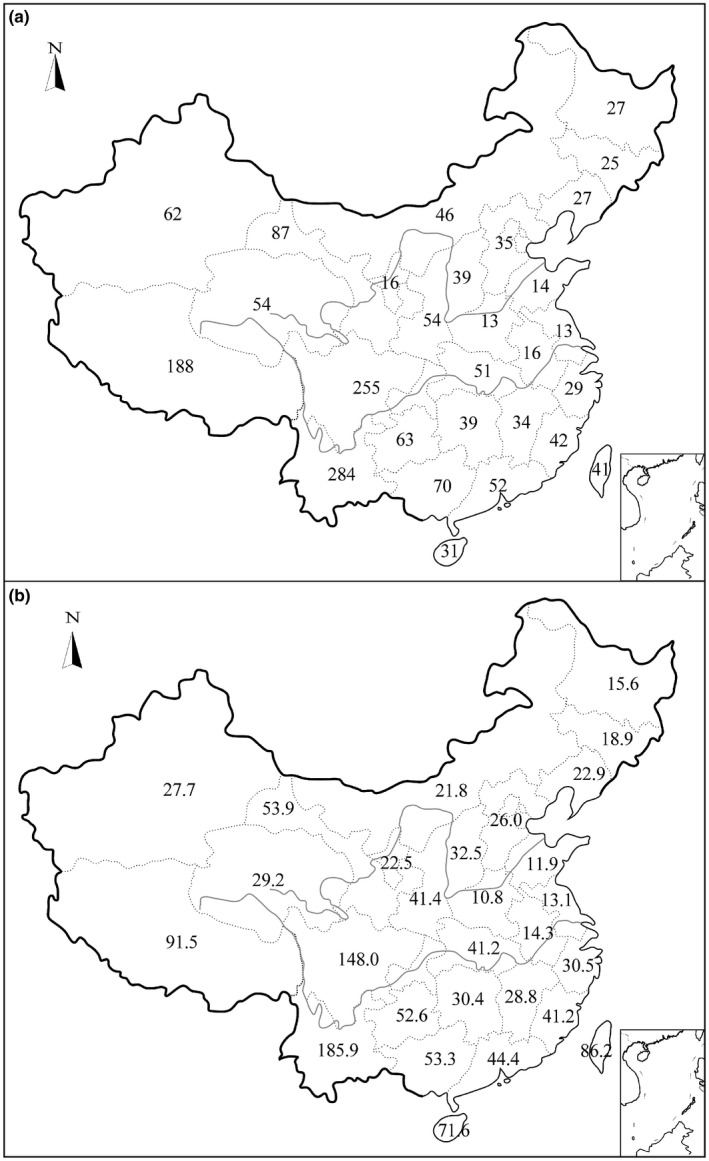
Distribution of species richness (a) and area‐corrected species richness (b) of parasitic plants in 28 provinces of China

The number of parasitic plant species varied largely among provinces, with the highest in the southwest and lowest in the northeast of China (Figure 1a). Yunnan was the highest in the number of parasitic species (*n* = 284), followed by Sichuan (*n* = 255) and Xizang (*n* = 188); Jiangsu (*n* = 13) and Henan (*n* = 13) were the lowest in the species number.

Similarly, the area‐corrected species richness of parasitic plants showed considerable variation among provinces, ranging from the lowest in Henan (10.8) to the highest richness in Yunnan (185.9; Figure 1b). It generally decreased from south to north and peaked in southwestern China.

**Figure 2:**
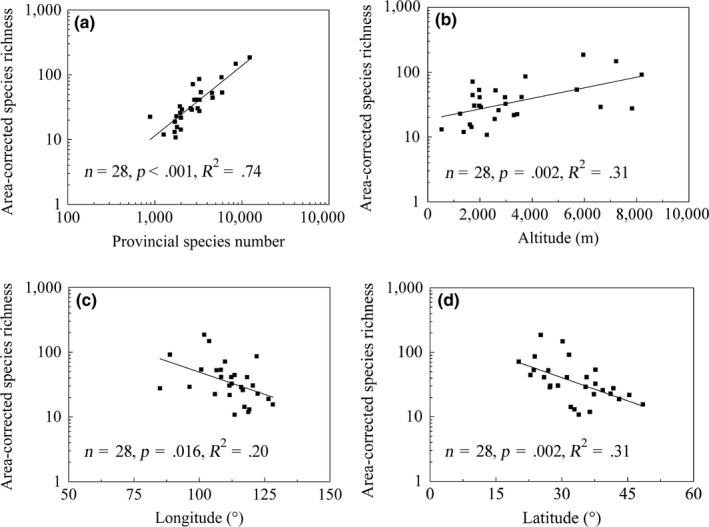
Linear regressions for log‐scale area‐corrected species richness of parasitic plants relative to log‐scale provincial species number (a), altitude (b), mid‐longitude (c), and mid‐latitude (d) in 28 provinces of China. Log‐scaling of the species number was used in the figure

Area‐corrected species richness of parasitic plants was positively correlated with the total species (including nonparasitic species) richness (*p *<* *.001, *R* ^2^ = .74). Moreover, it increased with increasing altitude (*p *=* *.002, *R* ^2^ = .31) but with decreasing longitude (*p* = .016, *R* ^2^ = .20) and latitude (*p* = .002, *R* ^2^ = .31; Figure 2).

Results of multiple regression analysis showed that altitude accounted for the most of variation in area‐corrected species richness among provinces, followed by latitude and longitude (Table 4).

**Table 4:** Multiple regression analyses showing the influence of the key environmental factors on area‐corrected species richness of parasites for 28 provinces in China (*n* = 28)

Dependent variable	Variable	*B* (±*SE*)	β	*t*	*p*
Area‐corrected species richness of parasites	(Constant)	−184.132 ± 103.453		−1.780	.088
	Altitude(m)	0.022 ± 0.004	1.140	5.183	.000
	Latitude(°)	−3.523 ± 0.669	−0.636	−5.264	.000
	Longitude(°)	2.468 ± 0.866	0.626	2.849	.009

*B*, Partial regression coefficients; β, Standardized regression coefficients; *t* statistics, and associated *p*‐values.

## 4 DISCUSSION

### 4.1 Floristic richness

To the best of our knowledge, this study is the first reporting the nationwide diversity and distribution of parasitic plant species in China. There are only several previous studies recording the species list for specific taxa or regions. For example, Han, Zhang, Hao, and Qiu (2002) collated a checklist of *Viscum* (Santalaceae) containing 12 species after consulting existing references and checking herbarium specimens. Wang, Tang, Xia, and Zhang (2007) provided a checklist of *Pedicularis* (Orobanchaceae), which included 181 species in Sichuan Province (including Chongqing) based on literature reviewing. Li, Wang, and Li (2002) introduced the floristic characteristics and biogeography of *Pedicularis* in Yunnan Province. Many parasitic plant species are harmful to crop plants or helpful to human health as traditional Chinese medicines (Guo et al., 2016; Li, 1998; Zhang et al., 2016). A complete floristic inventory of parasitic plants provides basic information for building control or conservation strategies to effectively managing these parasitic plants.

Our study can be also of international use. We recorded 678 parasitic plant species, accounting for 2.28% of China's total angiosperm species; this proportion is much higher than the value worldwide (1%, Těšitel, 2016), also higher than that of Turkey that has 146 parasitic angiosperms, corresponding to 1.29% of its flora (Sürmen et al., 2015), but slightly lower than that of Nepal that has 151 parasitic species, occupying 2.93% of its total angiosperm species (Joshi, Joshi, & Joshi, 2000; O'Neill & Rana, 2016). Importantly, the endemism is pronounced in China's parasitic flora, with almost two‐thirds species endemic to China. This figure is much higher than that of Turkey (13.01%) and Nepal (8.61%). This high proportion of endemism may be ascribed to the extremely high richness of native plant species in China, which is often assumed to result from the diversity of climate and topography. From south to north, Chinese vegetation covers tropical rainforest, subtropical evergreen forest, temperate deciduous forest, and boreal forest, spanning several climate zones (Wu, 1980). From east to west, Chinese topography is characterized by terrain, ranging from plains in the eastern provinces (>500 m at altitude) to basins in the middle ones (1,000–2,000 m at altitude), to the Qinghai‐Tibet Plateau in the west provinces (above 4,000 m at altitude; ECCPG, 1985b). Such a climate and geographic diversity may result in a wide range of habitats and facilitate local species differentiation, giving rise to endemism (Huang, Ma, & Huang, 2017; Wen et al., 2016). For example, the high species richness of the genus *Pedicularis* (including 441 herbaceous species and 89 subspecies or variants) in China (Wu et al., 1994–2012) has been frequently attributed to the extremely high topographic heterogeneity, mainly resulting from the uplift of the Tibetan Plateau, in the southwest mountainous areas (Zhao et al., 2016).

### 4.2 Life forms

Perennial herbs predominate the recorded parasitic plants in China. This is presumably because these herb species may have a short annual growing period but can accumulate biomass for several years to produce seeds and complete life histories, which allows them to use abundance ephemeral resource of host plants (Wu, 1980). Most of China is in temperate zone, where growth of host plants is often season dependent, providing seasonal varied resources available for parasitic plants. The activities of parasitic plants are physiologically dependent on the growth rhythm of their host plants. Even in southwest provinces of China, herbaceous plants also account for a large part of their parasitic flora (e.g., 76.76% in Yunnan and 87.84% in Sichuan). This is partly because these parasitic plants are mostly living in mountain and subalpine areas, where the winter is often long and frigid, leading to a short annual growth period. For example, of the highly diversified *Pedicularis*, 151 species are found in the alpine areas of southwest China (Li et al., 2002).

Among the four categories of parasitic plants, more than 3/4 is root hemiparasites. This figure is close to the proportion in Nepal, in which 108 of the total 151 parasitic plant species are root hemiparasites; indeed, at the global scale, it is estimated that more than half of the total parasites is root hemiparasites. The predominance of root hemiparasites may be ascribed to the fact that stable underground soil temperature and moisture (relative to the aboveground microclimate) facilitate plant growth and that they can be avoid of aboveground herbivores. More importantly, root hemiparasites are able to obtain more sunlight and limited resources in poor‐nutrition habitats than in rich‐nutrition habitats, thus making them have higher competitive ability over other parasitic types (Dueholm et al., 2017; Press & Phoenix, 2005). Additionally, it is also possible that the dispersal of root hemiparasites could be more advantageous than stem ones because seeds transported with soil are more likely to locate their hosts than those transported by animals for stem parasites.

### 4.3 Floristic distribution

We have shown that parasitic plants widely distribute throughout different regions of China, spanning across different biomes. This is consistent with the extensive distribution on a global scale, ranging from the tropics to the Arctic. Such a wide distribution is associated with the fact that parasitic plants are a diverse group of angiosperms with regard to their morphology, taxonomy, and phylogeny, which enable them to adapt various habitats (Press & Phoenix, 2005). However, the distribution of species richness is not even among provinces. For example, the richness is particularly high in the southwest China, but is extremely low in the northwest and northeast provinces. Such a distribution is consistent with the prediction of host‐quality hypothesis (Watson, 2009), which claims that parasites should present a clumped distribution.

Indeed, the distribution pattern of parasitic plants is similar to that of nonparasitic higher plants, especially of endemic plants in China (Huang et al., 2011), as indicated by the close across‐province relationship between the total species richness and parasitic plant richness revealed in our study. This positive relationship could be because high host diversity can foster parasites diversity (Hechinger & Lafferty, 2005). In fact, the geographic distribution pattern of Chinese parasitic plants can also be explained with climate and geomorphological conditions, as for the nonparasitic plants of China. We have found negative relationships of area‐corrected parasitic species richness with longitude and latitude. Across China, mean annual temperature decreases with increasing latitude from south to north, and mean annual precipitation generally decreases with the decreasing longitude from east to west except for the south part. Moreover, mountains dominate the geomorphological area of southwest China, which provides various habitats for plant living, such that altitude and the species richness of parasitic plants are positively associated. Additionally, a wide elevational range resulting from high mountains and deep canyons may cause high habitat heterogeneity, possibly improves local species diversity in the area. Therefore, higher plant species diversity peaks in the southwest China, which is also hotspot of plant species diversity worldwide. Likewise, the parasitic plant diversity also peaks in southwestern provinces of China. In particular, we have shown that altitude explained most of variation of area‐corrected species richness among provinces. This is similar to the results of recent studies showing that altitude is a significant factor influencing spatial pattern of species richness (Liu, Zheng, & Gong, 2017). Zhu, Kang, Jiang, and Liu (2008) even found that altitude accounted for more than one‐third of the variances of species richness patterns of forest communities in Mountain Helan, northwestern China. This could be because altitude influences both climate and geomorphology, and hence, it is more likely to be indicator than longitude and latitude for the species distribution of parasitic plants.

In summary, our study for the first time provides a comprehensive checklist of China's parasitic angiosperms, which cover 2.28% of Chinese angiosperms and are mostly endemic to China. Of the recorded parasites, perennial herbs and root hemiparasites are the predominating life forms. Moreover, the distribution of species richness is heterogeneous and the richness is the highest in the southwest of China, similar to the distribution pattern for Chinese angiosperms. The leading factors responsible for distribution include latitude, longitude, and altitude, of which altitude accounts for most of variation in specie richness among provinces. In conclusion, our results indicate that China is rich in parasitic plant species and their distribution is generally similar to overall distribution pattern of Chinese angiosperms. We suggest that the positive relationship between nonparasitic and parasitic plant species richness deserves future studies.

## CONFLICT OF INTEREST

None declared.

## DATA ACCESSIBILITY

Data available from the Dryad Digital Repository.

## AUTHOR CONTRIBUTIONS

G. Z. and S. S. conceived the ideas; G. Z. and Q. L. collected the data; Q. L. and G. Z. analyzed the data; and G. Z. and S. S. led the writing.

## References

[ece33992-bib-0001] APG IV . (2016). An update of the Angiosperm Phylogeny Group classification for the orders and families of flowering plants: APG IV. Botanical Journal of the Linnean Society, 181, 1–20.

[ece33992-bib-0002] Bardgett, R. D. , Smith, R. S. , Shiel, R. S. , Peacock, S. , Simkin, J. M. , Quirk, H. , & Hobbs, P. J. (2006). Parasitic plants indirectly regulate below‐ground properties in grassland ecosystems. Nature, 439, 969–972. https://doi.org/10.1038/nature04197 16495998 10.1038/nature04197

[ece33992-bib-0003] Bellot, S. , & Renner, S. S. (2013). Pollination and mating systems of Apodanthaceae and the distribution of reproductive traits in parasitic angiosperms. American Journal of Botany, 100, 1083–1094. https://doi.org/10.3732/ajb.1200627 23703856 10.3732/ajb.1200627

[ece33992-bib-0004] Bromham, L. , Cowman, P. F. , & Lanfear, R. (2013). Parasitic plants have increased rates of molecular evolution across all three genomes. BMC Evolutionary Biology, 13, 1–11. 23782527 10.1186/1471-2148-13-126 PMC3694452

[ece33992-bib-0005] Brown, J. K. M. , & Tellier, A. (2011). Plant‐parasite coevolution: Bridging the gap between genetics and ecology. The Annual Review of Phytopathology, 49, 345–367. https://doi.org/10.1146/annurev-phyto-072910-095301 10.1146/annurev-phyto-072910-095301 21513455

[ece33992-bib-0006] Calatayud, J. , Hórreo, J. L. , Madrigalgonzález, J. , Migeon, A. , Rodríguez, M. Á. , Magalhães, S. , & Hortal, Joaquín (2016). Geography and major host evolutionary transitions shape the resource use of plant parasites. Proceedings of the National Academy of Sciences of the United States of America, 113, 9840–9845. https://doi.org/10.1073/pnas.1608381113 27535932 10.1073/pnas.1608381113 PMC5024629

[ece33992-bib-0007] Ding, B. , Li, G. , Fu, C. , & Yang, S. (2010). Flora of Tianmushan (vols. 2–4). Hangzhou, China: Zhejiang University Press.

[ece33992-bib-0008] Dueholm, B. , Bruce, D. , Weinstein, P. , Semple, S. , Møller, B. L. , & Weiner, J. (2017). Spatial analysis of root hemiparasitic shrubs and their hosts: A search for spatial signatures of above‐and below‐ground interactions. Plant Ecology, 218, 185–196. https://doi.org/10.1007/s11258-016-0676-8

[ece33992-bib-0009] Editorial Committee of China's Physical Geography (ECCPG) (1985a). China's physical geography (pandect). Beijing, China: Science Press.

[ece33992-bib-0010] Editorial Committee of China's Physical Geography (ECCPG) (1985b). China's physical geography. Beijing, China: Science Press.

[ece33992-bib-0011] Editorial Committee of Flora Reipublicae Popularis Sinicae (ECFRPS) (1959–2004). Flora Reipublicae Popularis Sinicae. Beijing, China: Science Press.

[ece33992-bib-0012] Fernández‐Aparicio, M. , Reboud, X. , & Gibot‐Leclerc, S. (2016). Broomrape weeds. Underground mechanisms of parasitism and associated strategies for their control: A review. Frontiers Plant Science, 7, 135 https://doi.org/10.3389/fpls.2016.00135 10.3389/fpls.2016.00135 PMC4759268 26925071

[ece33992-bib-0013] Grewell, B. J. (2008). Parasite facilitates plant species coexistence in a coastal wetland. Ecology, 89, 1481–1488. https://doi.org/10.1890/07-0896.1 18589512 10.1890/07-0896.1

[ece33992-bib-0014] Guo, Y. , Cao, L. , Zhao, Q. , Zhang, L. , Chen, J. , Liu, B. , & Zhao, B. (2016). Preliminary characterizations, antioxidant and hepatoprotective activity of polysaccharide from *Cistanche deserticola* . International Journal of Biological Macromolecules, 93, 678–685. https://doi.org/10.1016/j.ijbiomac.2016.09.039 27637449 10.1016/j.ijbiomac.2016.09.039

[ece33992-bib-0015] Han, R. , Zhang, D. , Hao, G. , & Qiu, H. (2002). Geographical distribution of Chinese species of *Viscum* (Viscaceae) and its hosts. Journal of Tropical and Subtropical Botany, 10, 222–228.

[ece33992-bib-0016] Hartley, S. E. , Green, J. P. , Massey, F. P. , Press, M. C. P. , Stewart, A. J. A. , & John, E. A. (2015). Hemiparasitic plant impacts animal and plant communities across four trophic levels. Ecology, 96, 2408–2416. https://doi.org/10.1890/14-1244.1 26594698 10.1890/14-1244.1

[ece33992-bib-0017] Hechinger, R. F. , & Lafferty, K. D. (2005). Host diversity begets parasite diversity: Bird final hosts and trematodes in snail intermediate hosts. Proceedings of Royal Society B, 272, 1059–1066. https://doi.org/10.1098/rspb.2005.3070 10.1098/rspb.2005.3070 PMC1599879 16024365

[ece33992-bib-0018] Heide‐Jørgensen, H. S. (2008). Parasitic flowering plants. Leiden, The Netherlands: Brill https://doi.org/10.1163/ej.9789004167506.i-438

[ece33992-bib-0019] Huang, J. , Chen, J. , Ying, J. , & Ma, K. (2011). Features and distribution patterns of Chinese endemic seed plant species. Journal of Systematics and Evolution, 49, 81–94. https://doi.org/10.1111/j.1759-6831.2011.00119.x

[ece33992-bib-0020] Huang, J. , Ma, K. , & Chen, B. (2014). Diversity and geographic distribution of endemic species of seed plants in China. Beijing: Higher Education Press.

[ece33992-bib-0021] Huang, J. , Ma, K. , & Huang, J. (2017). Species diversity distribution patterns of Chinese endemic seed plants based on geographical regions. PLoS ONE, 12, e0170276 https://doi.org/10.1371/journal.pone.0170276 28114417 10.1371/journal.pone.0170276 PMC5256866

[ece33992-bib-0022] Joel, D. M. , Gressel, J. , & Musselman, L. J. (2013). Parasitic Orobanchaceae: Parasitic mechanisms and control strategies. Berlin: Springer https://doi.org/10.1007/978-3-642-38146-1

[ece33992-bib-0023] Joshi, A. R. , Joshi, D. P. , & Joshi, K. (2000). Status of some endemic plants in Nepal. Tiger Paper, 27, 15–20.

[ece33992-bib-0024] Kavanagh, P. H. , & Burns, K. C. (2012). Mistletoe macroecology: Spatial patterns in species diversity and host use across Australia. Biological Journal of the Linnean Society, 106, 459–468. https://doi.org/10.1111/j.1095-8312.2012.01890.x

[ece33992-bib-0025] Li, Y. (1998). Weed flora of China. Beijing: China Agriculture Press.

[ece33992-bib-0026] Li, D. , & Ding, Y. (2005). Distribution, present situation and conservation strategy of the genus *Phacellaria* . Biodiversity Science, 13, 262–268. https://doi.org/10.1360/biodiv.040204

[ece33992-bib-0027] Li, J. , & Dong, M. (2011). Impacts of plant parasitism on structure and function of ecosystems. Acta Ecologica Sinica, 31, 1174–1184.

[ece33992-bib-0028] Li, W. , Wang, H. , & Li, D. (2002). Biogeography and species diversity of *Pedicularis* (Scrophulariaceae) of Yunnan. Acta Botanica Yunnanica, 24, 583–590.

[ece33992-bib-0029] Liu, Q. (2013–2016). Flora of Jiangsu (vols. 2–5). Nanjing, China: Phoenix Science Press.

[ece33992-bib-0030] Liu, K. , Zheng, Z. , & Gong, D. (2017). Elevational patterns of species richness and their underlying mechanism. Chinese Journal of Ecology, 36, 541–554.

[ece33992-bib-0031] Luo, Y. , Sui, Y. , Gan, J. , & Zhang, L. (2015). Host compatibility interacts with seed dispersal to determine small‐scale distribution of a mistletoe in Xishuangbanna, Southwest, China. Journal of Plant Ecology, 9, 77–86.

[ece33992-bib-0032] McNeal, J. R. , Arumugunathan, K. , Kuehl, J. V. , Boore, J. L. , & dePamphilis, C. W. (2007). Systematics and plastid genome evolution of the cryptically photosynthetic parasitic plant genus *Cuscuta* (Convolvulaceae). BMC Biology, 5, 55 https://doi.org/10.1186/1741-7007-5-55 18078516 10.1186/1741-7007-5-55 PMC2242782

[ece33992-bib-0033] Naumann, J. , Salomo, K. , Der, J. P. , Wafula, E. K. , Bolin, J. F. , Maass, E. , … Wanke, S. (2013). Single‐copy nuclear genes place haustorial Hydnoraceae within Piperales and reveal a Cretaceous origin of multiple parasitic angiosperm lineages. PLoS ONE, 8, e79204 https://doi.org/10.1371/journal.pone.0079204 24265760 10.1371/journal.pone.0079204 PMC3827129

[ece33992-bib-0034] O'Neill, A. R. , & Rana, S. K. (2016). An ethnobotanical analysis of parasitic plants (Parijibi) in the Nepal Himalaya. Journal of Ethnobiology and Ethnomedicine, 12, 14–28. https://doi.org/10.1186/s13002-016-0086-y 26912113 10.1186/s13002-016-0086-y PMC4765049

[ece33992-bib-0035] Poulin, R. (2011). The many roads to parasitism: A tale of convergence In Rollinson D., & Hay S. I. (Eds.), Advances in parasitology, Vol. 74 (pp. 1–40). Burlington: Academic Press. 10.1016/B978-0-12-385897-9.00001-X 21295676

[ece33992-bib-0036] Press, M. C. , & Phoenix, G. K. (2005). Impacts of parasitic plants on natural communities. New Phytologist, 166, 737–751. https://doi.org/10.1111/j.1469-8137.2005.01358.x 15869638 10.1111/j.1469-8137.2005.01358.x

[ece33992-bib-0037] Rejmánek, M. , & Randall, J. M. (1994). Invasive alien plants in California: 1993 summary and comparison with other areas in North America. Madroño, 41, 161–177.

[ece33992-bib-0038] Santos, J. C. , Nascimento, A. R. T. , Marzinek, J. , & Leiner, N. (2017). Distribution, host plants and floral biology of the root holoparasite *Langsdorffia hypogaea*, in the Brazilian savanna. Flora, 226, 65–71. https://doi.org/10.1016/j.flora.2016.11.008

[ece33992-bib-0039] Sauerborn, J. , Mullerstover, D. , & Hershenhorn, J. (2007). The role of biological control in managing parasitic weeds. Crop Protection, 26, 246–254. https://doi.org/10.1016/j.cropro.2005.12.012

[ece33992-bib-0040] Stewart, G. R. , & Press, M. C. (1990). The physiology and biochemistry of parasitic angiosperms. Annual Review of Plant Biology, 41, 127–151. https://doi.org/10.1146/annurev.pp.41.060190.001015

[ece33992-bib-0041] Sürmen, B. , Kutbay, H. G. , & Yilmaz, H. (2015). Parasitic angiosperm plants of Turkey. Journal of the Institute of Science & Technology, 5, 17–24.

[ece33992-bib-0042] Těšitel, J. (2016). Functional biology of parasitic plants: A review. Plant Ecology & Evolution, 149, 5–20.

[ece33992-bib-0043] Těšitel, J. , Mládek, J. , Horník, J. , Těšitelová, T. , Adamec, V. , & Tichý, L. (2017). Suppressing competitive dominants and community restoration with native parasitic plants using the hemiparasitic *Rhinanthus alectorolophus* and the dominant grass *Calamagrostis epigejos* . Journal of Applied Ecology, 54, 1487–1495.

[ece33992-bib-0044] Wang, L. , Jia, Y. , Zhang, X. , & Qin, H. (2015). Overview of higher plant diversity in China. Biodiversity Science, 23, 217–224. https://doi.org/10.17520/biods.2015049

[ece33992-bib-0045] Wang, J. , Tang, Y. , Xia, Y. , & Zhang, L. (2007). Geographical pattern of species richness of *Pedicularis* (Scrophulariaceae) in Sichuan and Chongqing and its relationship with main environmental factors. Acta Botanica Yunnanica, 29, 51–57.

[ece33992-bib-0046] Watson, D. M. (2009). Determinants of parasitic plant distribution: The role of host quality. Botany‐Botanique, 2009(87), 16–21. https://doi.org/10.1139/B08-105

[ece33992-bib-0047] Weber, E. , Sun, S. G. , & Li, B. (2008). Invasive alien plants in China: Diversity and ecological insights. Biological Invasions, 10, 1411–1429. https://doi.org/10.1007/s10530-008-9216-3

[ece33992-bib-0048] Wen, Z. , Quan, Q. , Du, Y. , Lin, X. , Ge, D. , & Yang, Q. (2016). Dispersal, niche, and isolation processes jointly explain species turnover patterns of nonvolant small mammals in a large mountainous region of China. Ecology & Evolution, 6, 946–960. https://doi.org/10.1002/ece3.1962 26941938 10.1002/ece3.1962 PMC4761768

[ece33992-bib-0049] Westwood, J. H. , Yoder, J. I. , Timko, M. P. , & Depamphilis, C. W. (2010). The evolution of parasitism in plants. Trends in Plant Science, 15, 227–235. https://doi.org/10.1016/j.tplants.2010.01.004 20153240 10.1016/j.tplants.2010.01.004

[ece33992-bib-0050] Wu, Z. (1980). The vegetation of China. Beijing, China: Science Press.

[ece33992-bib-0051] Wu, Z. , Raven, P. H. , & Hong, D. (1994–2012). Flora of China. Beijing: Science Press.

[ece33992-bib-0052] Xing, Y. , Zhang, C. , Fan, E. , & Zhao, Y. (2016). Freshwater fishes of China: Species richness, endemism, threatened species and conservation. Diversity & Distributions, 22, 358–370. https://doi.org/10.1111/ddi.12399

[ece33992-bib-0053] Xue, W. (2011). Statistical analysis and SPSS application, 2nd ed. Beijing, China: Publishing House of Electronics Industry.

[ece33992-bib-0054] Zhang, G. (2007a). Plant biodiversity of Banqiao Natural Reserve in Anhui province. Nanjing, China: Nanjing Normal University Press.

[ece33992-bib-0055] Zhang, X. (2007b). Vegetation map of the People's Republic of China (1:1000000) and its illustration put to press. Beijing, China: Geology Press.

[ece33992-bib-0056] Zhang, M. , Chen, Y. , Ouyang, Y. , Huang, Z. , Silva, J. A. T. D. , & Ma, G. (2015). The biology and haustorial anatomy of semi‐parasitic *Monochasma savatieri* Franch. ex Maxim. Plant Growth Regulation, 75, 473–481. https://doi.org/10.1007/s10725-014-0010-1

[ece33992-bib-0057] Zhang, L. , Zhao, Y. , Wang, Z. A. , Wei, K. , Qiu, B. , Zhang, C. , … Li, M. (2016). The genus *Boschniakia* in China: An ethnopharmacological and phytochemical review. Journal of Ethnopharmacology, 194, 987–1004. https://doi.org/10.1016/j.jep.2016.10.051 27773803 10.1016/j.jep.2016.10.051

[ece33992-bib-0058] Zhao, L. , Li, J. , Liu, H. , & Qin, H. (2016). Distribution, congruence, and hotspots of higher plants in China. Scientific Reports, 6, 19080 https://doi.org/10.1038/srep19080 26750244 10.1038/srep19080 PMC4707485

[ece33992-bib-0059] Zhu, Y. , Kang, M. , Jiang, Y. , & Liu, Q. (2008). Altitudinal pattern of species diversity in woody plant communities of Mountain Helan, northwestern China. Journal of Plant Ecology, 32, 574–581.

[ece33992-bib-0060] Zwanenburg, B. , Mwakaboko, A. S. , & Kannan, C. (2016). Suicidal germination for parasitic weed control. Pest Management Science, 72, 2016–2025. https://doi.org/10.1002/ps.4222 26733056 10.1002/ps.4222

